# An exploration of context and learning in endurance sports coaching

**DOI:** 10.3389/fspor.2023.1147475

**Published:** 2023-04-17

**Authors:** Andrew Kirkland, Joe Cowley

**Affiliations:** ^1^ Faculty of Health Sciences and Sport, University of Stirling, Stirling, United Kingdom; ^2^ Faculty of Education, University of Stirling, Stirling, United Kingdom

**Keywords:** triathlon, sport pedagogy research, cycling, remote coaching, running, online learning

## Abstract

**Introduction:**

This study explored contextual factors which influence coach learning of an international cohort of endurance sports coaches.

**Methods:**

Following ethical approval, 839 coaches, 612 coached athletes and 8,352 non-coached athletes participated in the research. A critical realist research philosophy was adopted, in which self-completion surveys were developed in consultation with coaches and industry end-users.

**Results and Discussion:**

The context was dominated by remote coaching practices and digital technology which shaped how coaches learn and thus, what it meant to be a coach. Unmediated learning sources were biophysically biased and largely delivered through marketised platforms designed to sell products. The study findings have broader implications within sport and education, in which it is suggested that remote coaching and learning platforms may sometimes create a sense of psycho-emotional detachment in which capacity for learning can be limited.

## 1. Introduction

What coaches do in their coaching process, and who they do it with, is fundamental to how and what they learn (1). The constructivist model of Hattie and Donoghue (2) is important in this regard in which they suggest the skill, will and thrill of the learner shapes their development. In a coaching context, learning skills, orientations towards learning and motivations shape how coaches learn. The historical and cultural context in which they operate will influence what coaches want to learn about (3, 4). Whilst there is evidence of a recent shift towards learner-centred processes (5), mediated learning programmes (e.g., coaching courses and clinics) and research often fails to recognise the learning demands of coaches (3, 6, 7) by failing to consider context (8). In endurance sport, coaches often use remote and digital coaching practices and their unmediated learning practices. This is because endurance athletes often training independently without the need for specific facilities and in places where coaches cannot readily observe performance. However, this coaching context or its influence on how coaches learn has not been investigated.

This research adds to knowledge by exploring the role and learning of endurance coaches internationally and how sports specific contextual factors, such as technology and market forces, influence their practice. It is not the aim of the study to redefine, provide a new model of coach learning or the coaching process. This is done so in the belief that sports coaching is not reducible to simple description or explanation (7). Rather, it is to present a Critical Realist (CR) and subjective perspective of how context shapes the learning of endurance coaches in an unmediated digital world, dominated by the internet and technology.

## 2. Original research

### 2.1. Coach learning

Whilst there are comprehensive texts such as Nelson, Groom and Potrac (9) and a growing body of literature which surrounds coach learning, few authors define what learning is or explore contextual factors that affect learning processes. However, we suggest that coach learning is a socially constructed phenomenon influenced by historical, economic, political and cultural forces (10, 11).

This suggestion is influenced by the work of Moon (12, 13) and Werthner and Trudel (14) who were instrumental in developing an understanding of how coaches learn, whether through mediated, unmediated or internal events. Moon's generic view of learning has been used extensively to conceptualise coach learning [e.g., (15–21)]. This non-reductionist model has been built on the premise that every concept relates to and modifies every other concept. Therefore, learning is complex and an all-encompassing theory of what is best does not exist. Moon's generic view of learning goes far beyond the simple conceptualisation and application of mediated, unmediated or internal learning opportunities.

Firstly, Moon recognises that her work primarily focusses on formal learning situations. However, most coach learning is simply a product of normal human behaviour and social interaction (12–14, 22). It tends to occur through trial-and-error, through experiences as an athlete, by watching other coaches and through self-directed learning, such as using the internet (14, 17, 23). Thus, coach learning can be viewed as the creation of knowledge through social participation in day-to-day practices (24) primarily in non-formal, situated and often unstructured events.

Secondly, Moon (13) highlights the importance of the emotional component of learning and how it is neglected by educational research and models of learning. She argues that for learning to occur, learners must firstly be willing to learn and then develop the ability to contextualise and apply information within the appropriate environment. Emotional engagement is important if deeper learning is to occur. Coaches are typically adult learners, who may only occasionally engage with tertiary education. When they do, they bring with them different learning dispositions (25), levels of experience and emotional “baggage”. As Hattie and Donoghue (2) state “The most important single factor influencing learning is what the learner already knows” (2). This knowledge affects not only their agency to learn, but also the consent they give to those who are charged with educating them (26). Thus, a “one-size fits all” approach to coach learning, which does not consider emotional engagement, is unlikely to be efficacious (27).

Finally, Moon (13) recognises that learning does not only occur in one direction. Rather, it can result in undesirable changes in behaviour or attitudes through assimilating with their environment, learning about outdated or ineffective practices, passively soaking up information from questionable sources or coaching without adequate reflection (28). Thus, some mediated learning situations can potentially result in learning that negatively influences coaching practice.

Thus, for learning to occur, something must change in the way that coaches currently think and/or act (13), and it is desirable for coaching practice to change for the better (29). Learning is an active process of knowledge construction rather than passive information consumption. Coach learning and practice are synergistic through the construction of interwoven beliefs, structures, practices and ideologies (10). Arguably, this results in complex discourses which are specific to the environment in which the coach operates. However, this sociocultural osmosis may mean that many coaches simply learn to maintain the status-quo of their coaching environment and ascribe to its pervading discourse (30) without deep reflection on their practices.

Mediated coach education, whist viewed as an important in raising the standards of coaching practice (9), can be relatively ineffective in facilitating learning [e.g., (9, 31–35). The reasons for this are complex and multi-faceted; however, a narrow disciplinary focus (15, 35, 36) on what is important to learn about, decided on by “experts” who may not wholly appreciate the learning context may be a contributory factor. Furthermore, coaches must also have sufficient agency to engage with learning (1) and consent to the “power” being wielded over them (37) by coach educators. Linked to this is socially mediated power relationships between coaches and coach educators, in which there can be resistance to change or intellectual reasoning (28, 38) from both sides. The fact that learning, culture and context are inherently relational (38, 39, 41) means that the agency of the learner must be considered when considering coach learning. “Planting the seeds” of intellectual and practical coaching competencies (42) requires recognition that at one extreme of the scale formal systematised coach education programmes have a tendency towards quasi-colonial quantification and control. This may deskill coaches (43) through promoting information consumption rather than knowledge construction (26). At the other extreme, the modus operandi of coaches may be the valorisation of wisdom of the other coaches and populist coaching information (44) without cognitive reasoning. The pragmatic middle-way is to recognise that coaches will do most of their learning in unmediated situations and internally.

Coach education moves slowly, in which frameworks are usually designed through positivistic and reductionist lens (8) and have typically result in “one-size” fits all curricula (6, 30, 45). The international (mediated) coach education landscape in endurance sport is diffuse and poorly defined. Whilst not reported in the literature, centrally funded National Governing Bodies (NGB) such as British Triathlon and British Cycling, whose coach education programmes were aligned to the UKCC framework, have in part been adopted by the World Governing Bodies, The Union Cycliste Internationale and World Triathlon. These programmes have been used to support nations without their own programmes. Throughout the world, most coach education programmes in endurance sport have been built on more traditional conceptualisations of face-to-face coaching, rather than reflecting needs within specific coaching contexts.

One of the motivations for conducting the present study was a belief that these curricula often bear little resemblance to the coaching context, they fail to consider what motivates coaches to learn and what they need to learn about to develop context specific competence. Lyle (46) highlights that this needs led approach can lead to the desire to conduct research based on a personal desire to improve practice. Adding to the provision of coach education by NGB's, TrainingPeaks, a commercial business, is the global leader in the provision of a digital platform to support endurance coaches. Their strategy has been typically to partner with larger NGB's internationally to encourage coaches to use their platform and these NGB's “outsource” technology related education to TrainingPeaks. They deliver “TrainingPeaks University” workshops and online courses, specifically focused on how to use their coaching Web technology, which is mainly reliant on the uploading of biophysical data but also supports the development of coach-athlete feedback loops.

Thus, coach-athlete interactions, political influences, governing bodies and market forces all shape learning processes (15, 47). In the digital age, different technologies have been incorporated into coaching and have shaped coaching practice, and opened new affordances for athletes seeking guidance, particularly in endurance sport. The “skill, will and thill” learning model of Hattie and Donoghue (2) is important in this regard. As adult learners, coaches will have domain specific beliefs (49) which influence how they interpret learning material in relation to context and relative to previous understandings shaped by pervading discourses in their sport (13).

Therefore, we suggest that it is vital to understand what coaches find interesting and influential on their learning. We also suggest that because the endurance coaching context has not previously been described, there is a limited evidence-base to support arguments for change at systemic level. However, coach education has tended to focus on a top down approach, providing coaching pathways which align to linear long-term athlete development models (LTAD), vested NGB interests and a drive towards elite performance. This is done without necessarily considering the learning wants and needs of coaches within their specific delivery contexts.

### 2.2. The sporting context

Endurance sport is a “broad church” in which athletes participate in events such as triathlon, running and cycling (49). Over the last 20 years or so, these sports have grown from being niche into a world-wide multi-billion-dollar industry (50). Whilst athletes on traditional LTAD pathways are important, growth has been driven by the emergence of participants defined as Personal Referenced Excellence (PRE) by Bailey et al. (51). They tend to be non-elite participants with personally constructed motivations such as completing challenging events including Ironman triathlon, marathon or a distance cycling events in personal best times.

The training environment in endurance sports affects the coaching process because it is less constrained by facilities than for many other sports. Athletes usually train independently on the roads, in the hills and in the oceans without coaching supervision. The popularity of mobile technology including portable GPS computers, power-meters and heart-rate monitors to collect a wealth of data in endurance athletes has increasing exponentially (52, 53) within endurance sport. These data primarily measure training load with a focus on frequency, duration and intensity (54). Athletes then upload data to online digital platforms such as Strava or TrainingPeaks for further analysis.

Athletes support an industry which has been created through multidimensional consumerism involving the selling of products such as sports equipment, event entries and sports nutrition products. The provision of coaching services also falls into this market (55). Forces acting within this market invariably influence what coaches wish to learn about. Many endurance athletes are self-coached; however, a small but important minority will engage the services of a coach to help them with their training (56).

In terms of technical performance, face-to-face coaching such as on the pool-deck, the running track or on a closed-road circuit is arguably very important. However, what endurance coaches do and how they do it, may be more driven by the athlete wants rather than needs. Whilst not confirmed by research, the coaching process is often far removed from traditional conceptualisations of coaching. Anecdotally, this results in more digital and remote methods of coaching in which the coach-athlete relationship becomes a transactional one where the coach delivers intangible services (57). These services are often dictated by market demands, i.e., the wants of their client and what the clients beliefs are surrounding the role of a coach. Consequently, such market demands, and other context specific discourses are likely to shape what coaches want to learn about. Additionally, the recent COVID-19 Global pandemic and subsequent lockdown has resulted in coaches outside the endurance sports domain using more online and digital coaching practices during the lockdown periods (58). Therefore, lessons learnt from endurance coaches operating in the digital world may have broader application to other sports and education.

### 2.3. Understanding context

Coach learning and practice involves ever shifting, complex and multifaceted processes (59, 60). These processes are influenced by social forces and discourses that have potential to legitimise or delegitimise certain ways of being (61). However, a Western discourse towards reductionism and simple aphoristic rules often results in research conducted through narrow theoretical disciplinary lenses, focused on readily measurable dimensions of coaching (8, 62). Such knowledge constructed through over-simplification invariably influences formal learning programmes. We argue that research should be sensitive to context and the often atheoretical nature of coaching, in which coaching behaviours are in-part emanations of the social structures in which they emerge. Doing so may lead to a fuller appreciation of the coaching process and context in which intertwined and inseparable factors represent their complexity. North (63) suggests that research requires a “space for an alternative approach to conceptualising coaching practice that seek to identify causal factors underpinning coaching outcomes” (63). Prior to seeing more interpretive and causal explanations, exploration and description of context is required. Furthermore, research questions should be co-created and designed through complementary practice with stakeholders operating within the context. Whilst theory construction has its place in this research process, it is important to recognise that a large and overlapping array of theories and components of theories can be used to influence applied practice (64, 65). To be impactful, research must also be “accepted, adopted and complied with by consumers such as endurance athletes, coaches and practitioners” (47) and presented in a way that makes sense to them.

### 2.4. Research aims

Therefore, the aim of this study is to explore the coaching context and learning of endurance coaches, specifically to “produce a very realistic account of [endurance] sport coaching which explores causal underpinnings” (7). A CR framing is employed with the intention to develop greater epistemological congruency between research and applied coaching practice. This study adds to the existing literature by potentially helping coach educators and the wider endurance coaching industry to better understand the market that they are operating within.

## 3. Materials and methods

### 3.1. Research perspective

A CR research ontology (7, 66, 64) was adopted with the explicit aim of contributing new knowledge that provides practical solutions to coaching problems within endurance sport. Fundamental to a CR ontology is the exploration and identification of causal tendencies rather than finding universal truths to explain complexity. Critical realists take a realist and subjective stance, believing that social structures can be real, but do not necessarily have a universal impact, and that they cannot necessarily be measured using simple empiricist methods (65). Critical Realists also posit that “the empiricist nature of causality is wrong” (69). The meaning of causality is philosophically contested, a debate that is covered by Groff (70). From a CR perspective, in an open-ecological-system, causation is complex, hard to define and emerges through the symbiotic relationship between social reality and human agency. Critical realists do not argue that positivistic and constructivist methods are unimportant in knowledge construction (61). Rather, data collected using such methods must be considered within the wider social content to which it applies. In the context of this study, this meant that mixed-methods were employed and that interpretation of the data was inherently realist, subjective and reflexive (8). The primary researcher has been embedded (7) in the endurance sports context as a coach, chartered scientist, coach educator and PRE-athlete with experience and expertise in the area. He is an “inside” observer whose perspective infused into the research process, helping with participant recruitment, informing on what questions were asked, how data were analysed (67, 68) and interpreted. As an actor immersed in the endurance world, knowledge, experience and judgement developed over time allows for deep understanding of these social realities and the potential causal mechanisms for them. This is consistent with a CR ontology in which hidden causal structures (61) are uncovered through in-depth research which can be facilitated by an insider perspective (69). However, such voice means that it is not possible to eliminate research biases (70, 71). This was balanced by the collection of objective data, which was interpreted through a subjective and realist lens. For trustworthiness and credibility purposes, reflexive processes were adopted, through discussions on interpretation with the second author, and in asking “critical friends” who are familiar with the coaching context to review a draft manuscript. These processes were vital to achieve the aim of the study, to produce a realistic account of the endurance context, which whilst familiar to an insider, goes further in exploring the complex causal underpinnings of context specific coach learning. Specifically, an intention of this research was to provide foundational description and interpretation of context. In doing so, our hope is that longitudinal type studies may emerge and from which deeper clues to causality can be explored. This approach is consistent with research model of Bishop (72) in which descriptive data is foundational for subsequent work to explore cause.

The CR approach in coaching allows for exploration of the complexities of the coaching context, which may provide a more realistic, accountable knowledge to coaching stakeholders (7). This interpretation of a CR ontology resulted in a bricolage approach being adopted to capture the context specific multi-layered, multi-faceted complexities of coaching (7, 73). The aim of doing so was to focus the mind on purposeful actions and what the intended consequences (37) of the research are. In other words, the intention was not to construct new theories or explore the data through a particular theoretical lens. Rather, it was to recognise the inherently complex and hard to define nature of coaching and to present a narrative that is useful to research end-users.

### 3.2. Survey design

A cross sectional, qualitative approach was used. The research was part of a wider study involving four surveys, with the data collected using the Online Survey platform. This approach was chosen as it is reported to provide rich qualitative data collection in a quick cost-effective manner. In this context, the use of surveys may generate comments that are more diverse than those observed in focus groups or individual one-to-one interviews [e.g., (78)].

These surveys were designed during project conceptualisation through the development of a stakeholder group led by the primary author. This group enabled us to gain a range of perspectives and opinions particularly on what questions to ask and their relevance to the context of stakeholders (76, 77). Whilst the approaches used were relatively consistent with a Delphi consensus method (78) they involved more informal, ad-hoc and organic processes, including consultation with sports coaches, consultants in coach development, data scientists and other research end-users involved in endurance sport, within the primary author's network. The final survey emerged through iterative refinement involving group members. Demographic and sources of knowledge questions were adapted from SportsCoachUK tracking study (79). These demographic questions were built upon using quantitative data from The State of Endurance Coaching Report (80), an unpublished industry report. Further elements of the survey were drawn from the themes identified by McCormick et al. (81) in relation to the psychological demands experienced by endurance athletes. This ad-hoc approach to survey design was used in recognition of the complexity of coaching and learning and the practicalities of conducting practical research. Themes for the questions related to demographics, coaching process, and learning.

Prior to being administered, the questionnaires were reviewed extensively for face and content validity by a by a panel of experts as aforementioned, including sport industry stakeholders. This was then piloted with a small group of coaches and athletes. Whilst adding to the complexity of the research process, such stakeholder engagement was viewed as fundamental to the research philosophy and study design (78). This co-creation added to challenges in study design, analysis and interpretation of data. However, it took priority over a narrower theoretical framing to add ecological validity and generalisability within relevant contexts.

### 3.3. Recruitment and procedures

Coaches and athletes were recruited for the study, in recognition that athlete perspectives, needs and wants influence coach learning and practice. There were two surveys for the coaches (1) a primarily quantitative survey, and (2) a primarily qualitative survey. With the first survey, we wanted to maximise completion rate through not making the survey too long. On the final page of this survey, there was a link to the 2nd qualitative survey, which could be completed by participants if they wished to. This was to avoid over-saturation and the practicalities of analysing large volumes of qualitative data. There was also a survey for athletes who had engaged an individual coach and one for non-coached athletes (including those coached within a club without individualised coaching support).

The study was approved by the relevant *University Ethics Committee* according to the *Declaration of Helsinki*. Following institutional approval, purposive sampling was employed where coaches and athletes were contacted through social media, in which the primary researcher used his links with industry, National Governing Bodies (NGB) and high-profile athletes to reach a broad international audience. Snowball sampling occurred (78) through sharing on social media. Data collection was completed prior to COVID-19.

### 3.4. Quantitative data

Quantitative data were downloaded from Online Survey and analysed within Microsoft Excel spreadsheets. Where data is presented as a Ranking Score, these were ordinal rankings which were numerically normalised to allow cross-comparison between participant groups. For example, responses of very important; important; not important; not important at all were assigned numerical values from +2 to −2, respectively, were summed for all responses provided and then expressed as a percentage of the maximum score possible.

### 3.5. Qualitative data

Qualitative data were downloaded from Online Survey and then organised and analysed using NVivo 11 (Qualitative Solution Research, 2017). A complementary approach was followed throughout the analyses. The primary author's expertise in endurance sport allowed him to immerse in the data, adding depth and richness to the data gathered (82). Themes were generated and developed from the data using the six stages of reflexive thematic analysis as a framework (83). Both inductive and deductive approaches were considered as these lend themselves to this revised reflexive approach (84).

This process started with the familiarisation of the data where the text were read and re-read. Initial codes were then generated using Nvivo 11 (Qualitative Solutions Research, 2017). Patterns were then identified amongst these codes to generate themes. These were then reviewed against the entire qualitative data set. Themes were then defined before writing up the data using the extracts that were relevant to the initial questions.

## 4. Results and discussion

The aim of this study was to explore the coaching context and learning of endurance coaches. The intention was not to provide irrefutable truths on endurance coaching practice (64) but to provide insight into an emerging and complex coaching context. By doing so, a substantial contribution to knowledge has been made by presenting a form of coaching that is different to how it has traditionally been conceptualised. This coaching has been shaped by a wider milieu, in which an inextricably, interconnected array of factors may influence coach and athlete interactions in the digital age.

### 4.1. Participant demographics

Participant demographics are often simply viewed as descriptive data. However, they provide context and are fundamental to implementing research into practice (72, 77). They also help in understanding societal and economic forces which shape the coaching context, coaching processes and coach learning. By segmenting demographics into different markets, “consumers” can be better understood and mechanisms for change can be more effectively developed (85). Whilst market segmentation has not typically been employed in coaching research, Sport England recently used market segmentation tools (86) to help stakeholders better understand the behaviours of different segments of society in the hope of growing participation levels. As part of research process, stakeholders were engaged with the intention of breaching the divide between research and applied practice (5, 62).

Nine thousand eight hundred and three participants completed the appropriate survey for their group (618 male and 198 female coaches; 362 male and 244 female coached athletes; 6,632 male and 1,516 female non-coached athletes). Average age was 44.2 ± 10.8 years (Coaches 44.0 ± 9.8 years; Coached athletes 41.0 ± 10.3 years; Non-coached athletes 44.4 ± 10.9 years). Forty-one percent of coaches were part-time, with another full-time job; 31% were full-time coaches; 18% were full-time coaches with another job; 6% were volunteers and 5% coached a few friends at a time. Forty-five percent of coaches worked independently; 27% worked independently but were also part of a wider coaching team/business/club; 23% worked within small team/business/club and 5% operated within a large team/business/club.

Coaches' income (reported in US$) was significantly lower than coached or non-coached athletes (*p* < 0.01) (Coaches $59510 ± $89877; Coached athletes $90988 ± $188243; Non-coached $104224 ± $337986) with substantial differences being apparent depending on where in the world participants lived. The USA, UK, Australia and New Zealand were the most prominent nations represented potentially due to two factors, that they represent key endurance sport markets and because surveys were only presented in English. Importantly, whilst there was considerable variation in income level, athletes tended fall within higher ABC1 income brackets. Further, participants tended to be educated to graduate and post-graduated educated levels as shown in Table 1. It should be noted that the non-coached athletes answers, whilst not necessarily addressing the research aims and objectives, contributed to understanding of the coaching context, demographics within the sport and provides clues surrounding the latent coaching market.

**Table 1:** Highest level of education between groups.

Level of qualification	Coaches (%)	Coached athletes (%)	Non-coached athletes (%)
Postgraduate	32	30	28
Higher education degree	29	28	30
Professional qualifications	21	17	17
Further education including vocational qualifications	9	9	
Doctorate	6	8	9
High school or secondary school	4	7	6

We were guided by the work of Bailey et al. (49), Collins and Collins (87) and the UK Sport Athlete Performance framework in determining athlete segments/categorisation for athletes. These segment categorisations terms were refined in consultation with stakeholders to ensure they were appropriate for an international audience and are shown in Table 2. The predominant segment for both coached and non-coached athletes was personal referenced excellence (PRE), identified as a group of adult athletes who are motivated to be the best they can be, but who may not necessarily have the talent, aspirations or agency to move to the next level (88). The data in Table 3 represents the relative percentage of athletes they coached in each segment, recognising that many coaches worked across several segments.

**Table 2:** Segments and types of athlete (ERE, elite referenced excellence; PRE, personal referenced excellence).

Segment	Type of athlete	Coaches (%)	Coached athletes (%)	Non-coached athletes (%)
ERE	Athletes who win on the world stage	4	2	<1
	Athletes with potential to win on the world stage in the next 4 years	8	2	< 1
PRE	Top level domestic athletes who perform at a high national level and/or world-leading age-groupers	14	23	9
	State or regional level athletes	16	11	9
	Age-group athletes focussed on achieving personal bests and/or qualification for prestigious events	26	52	55
Recreational	Athletes who participate for social, fun, fitness and personal challenge motives	19	9	25
Development	Junior development athletes aged 13-17 years	10	N/A	N/A
	Youth development athletes aged 12 years and below	4	N/A	N/A

**Table 3:** Main sport of participants.

Sport	Coaches (%)	Coached athletes (%)	Non-coached athletes (%)
Triathlon	29	50	31
Running	23	8	16
Road cycling	22	32	45
Off-Road Cycling (MTB/CX/Gravel)	11	7	6
Other Multi-sport (Duathlon; Swim-run; X-terra etc.)	9	1	1
Track Cycling	3	0	0
Other	2	1	0

The athlete demographic suggests that most are educated professionals who are highly driven and have demanding work/training/life schedules and family commitments (See Sport England segments). Whilst, the segments of Bailey et al. (49) give clues about the motivations of athletes, it is suggested that the wider sporting milieu will influence what expectations they have on a coach and how these expectations are interconnected with the coaching process. For example, the coach-athlete relationship within the PRE-segment can be a more of a transactional one of service provision rather than being a two-way relationship. Furthermore, group coached sessions are constrained by the availability of facilities such as swimming pools, running tracks and closed-road circuits for cycling. This is reflected in how athletes train, regardless of whether they are coached or not. Anecdotally, younger athletes, particularly those on long term athletic development (LTAD) pathways are far more likely to be coached in a traditional sense, in which coaches observe and feedback on performance.

Table 2 shows the main sports of the study participants. Interestingly, this data highlights that triathletes are more likely to be coached than cyclists or runners. Whilst the reasons for this phenomenon are unclear, triathlon is a relatively new sport in which tradition is less imbedded and culturally more open to coaching and innovation.

### 4.2. Coach education and learning

The relevance of coaching context: Is Technology Redefining the Coaching Process?

Cooper and Allen (89) suggest that coaching involves social interaction involving at least two people, a coach and an athlete. These interactions result in sharing of information to guide the actions of both actors (93) with the aim of improving the athlete's performance. The present research is important because we show that the coaching process, including planning, delivery of sessions and communication, is fundamentally shaped by technology without necessitating direct coach-athlete social-interaction. Table 4 shows that coaches spent very little time delivering traditional face-to-face coaching. However, the standard-deviation values show that there are large differences in the time spent on specific coaching tasks. This is likely to be because there is no “one-size-fits-all” conceptualisation of endurance coaching. Rather, we suggest it is a “cottage industry” in which coaches typically work independently, on a part-time basis and in a way that fits around their life demands and interests.

**Table 4:** Proportion of time coaching.

	Admin	Planning and prescribing	Remote coaching	Face-to-face coaching	At competitions	Travelling	Total
Mean (hours)	3.0	7.0	4.4	4.6	2.1	2.6	22.1
SD	3.3	5.9	4.3	6.3	2.7	3.4	16.0
% Total	12.8	33.5	19.4	19.6	6.7	8.0	100.0

Similarly, the income level and age of athlete PRE-athletes is consistent with the market segments from Sport England (86) of participants who are income rich and time poor. This is reflected in the fact that 70% of coached athletes train alone or in small groups (16%), with only 11% training in a club environment. From a coaching perspective, such unmediated training limits the possibilities for “real-time” feedback to assess the quality of training. Rather, the preferred method to develop feedback loops is through the integration of data collected using digital devices such as GPS, power-meters and heart-rate monitors which are uploaded to web platforms for analysis by the coach. This data constitutes the main type of feedback coaches ask for (Table 5), thus reducing training to a primarily metabolic event, with less focus on psychosocial, technical and tactical aspects of performance. Whilst there are exceptions, the coaching process is often reduced to online training prescription and data sharing. Whilst the reasons may be complex, it is suggested that technology is reducing coaching to a measurement-induced myopic process in which psychoemotional elements of performance could be neglected (60). A coach can prescribe additional technical content, performance criteria and psychological skills to practice, the athlete must be able to benchmark their performance against criteria established by the coach (94). Doing so requires advanced conceptualisation of the coaching process and the ability to foster an effective coach-athlete feedback loop. However, coaches in this study do not tend to rank these factors as important or interesting and many are unlikely to engage in such practices as a result.

**Table 5:** What feedback coaches ask athletes to track? (All that apply).

Feedback data	*N*
Training Load (Power; GPS; Heart rate etc.)	538
Training Load (Time)	426
Subjective feedback (mood; work-life balance)	312
Training Load (RPE)	235
Weight	168
Other metrics (e.g., Hours of sleep; % Body Fat; Menstruation; Motivation; Fatigue etc.)	162
Nutrition	118

**Figure 1:**
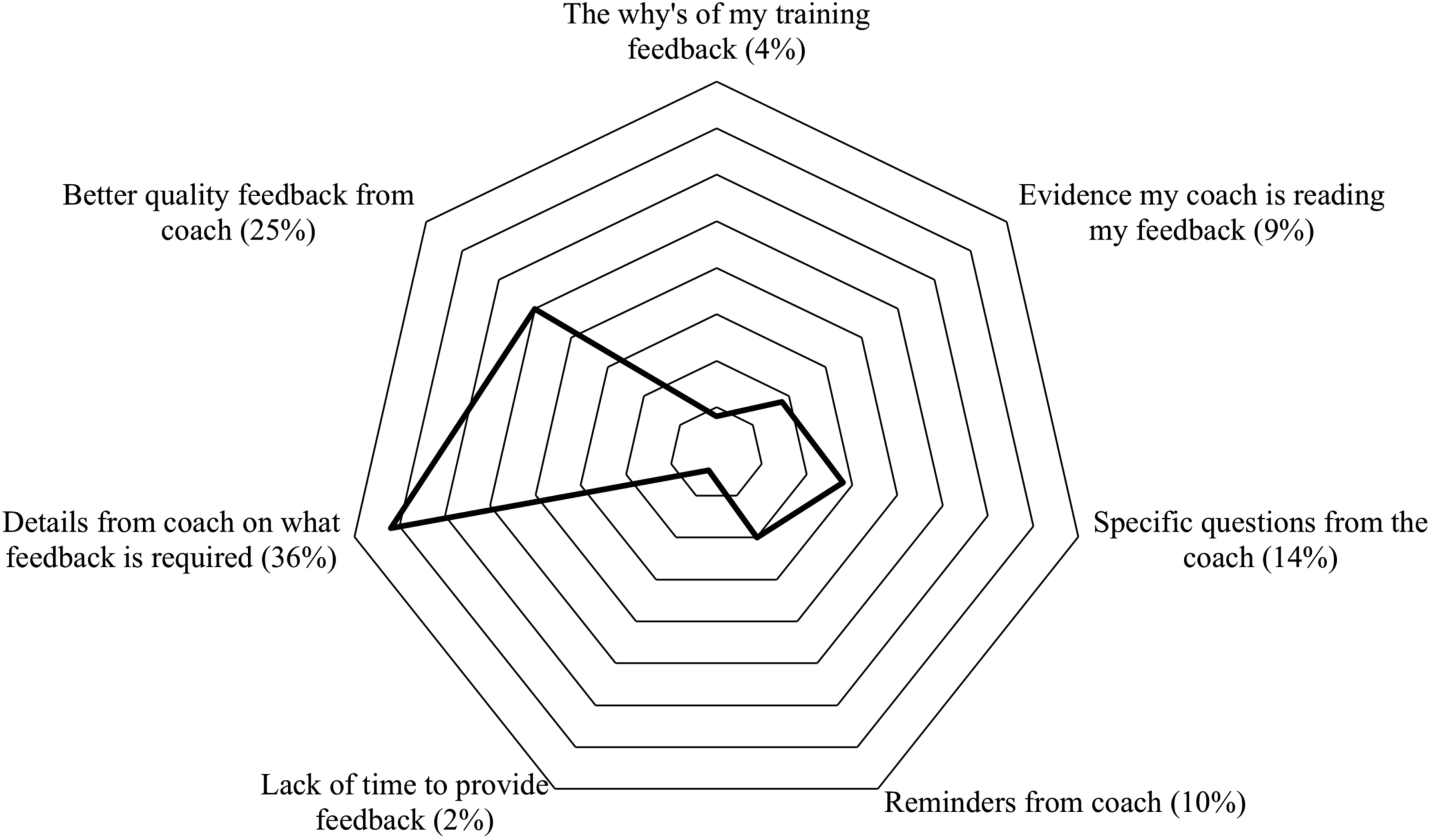
Reasons how coaches could help athletes provide better feedback.

Some may argue that training prescription is not coaching because it does not involve giving direct technical advice (91). Furthermore, without face-to-face contact, social interaction may be limited by asynchronous engagement using web feedback. This could mean that social rewards and emotions may be limited. This is reflected in coaching beliefs in which there is a common perception that athletes tend to be poor at providing qualitative feedback, an area that was discussed with stakeholders in the design of this project. One reason may be that data from many devices can be interfaced with and uploaded to the web automatically, but qualitative feedback requires athletes to take time to reflect “on action” (92). Whilst 33% of coached athletes believed that they provided high-quality feedback, the remainder recognised that they could do better. Using an open-ended question, athletes were asked how their coach could help them provide better feedback and seven themes were identified, as shown in Figure 1. These themes suggest that coaches need to develop skills in eliciting quality feedback, such as through questioning and being able to provide good-quality feedback themselves. In the digital environment, good quality qualitative feedback is essential to augment biophysical metrics and in building effective coach-athlete relationships. Such data replaces the coaches “eyes and ears” in the feedback loop. However, there is there is little precedent on how best to engage in meaningful coach-athlete dialogue and social connection in the endurance sport digital environment.

Humans learn through thinking and knowing, and such cognitive abilities are best developed through osmotic social-interaction and dialogue with others (93, 97). However, we are at an epoch in evolution because digital technology has revolutionised how people communicate, interact and learn. COVID-19 has accelerated these processes. Whilst we are “always connected”, Mezirow (95) suggests that adult learning is best achieved through communities which engage in rationale discourse and action. However, technology appears to present fewer opportunities for meaningful social interaction, in which self-reflection and critical thinking may be compromised. This is important within the context of this study in which coaches stated that learning through experience, experience as an athlete and reflecting on past coaching are most important to them in developing their coaching. However, Brookfield (96) suggests “it is enormously difficult to stand outside of one's own interpretive frameworks through an act of one's own mental volition”. If the coaching-process occurs primarily in front of a computer screen, then this is likely to have negative implications on reflection and reflexive learning. For example, there has been a tendency in educational settings to use technology as a new type of vessel for information transference, without considering the implications to and mechanisms of learning in such an environment (97). This challenge applies equally to remote coach-athlete relationships.

Whilst human interaction rather than virtual connection is vital to create and share learning experiences (93), the digital environment means that such interaction is often removed from the coaching process in an endurance sport context. Further, in a market driven economy, PRE-athletes have greater autonomy and fewer constraints in how they choose to be coached. If these athletes consider that prescription of training, the analysis of and subsequent feedback on training is coaching, then academic debates surrounding what coaching is or is not and the coaching process are inconsequential. Notwithstanding, traditional conceptualisations of what coaching is (91) have probably influenced what NGB's have delivered on their programmes. For example, to coach individual athletes in the UK, coaches typically needed a UKCC Level 3 qualification. These frameworks require coaches complete Level 1 and Level 2 qualifications which focused on face-to-face coaching and group session “design”. The data in Table 6 are interesting in this regard. Planning and prescribing endurance training was viewed as the most important thing to learn about for endurance coaches. Despite believing that online and desktop coaching software was viewed as more important than psychological elements of performance, coaches believed that coach education had poorly prepared them to use technology. Therefore, commercial providers such as TrainingPeaks, who have vested interests, have filled a gap in the market to support coaches with technological advances. From a neoliberal standpoint, we suggest that “the market” has greater influence over what is considered to be coaching, than academics or NGB's have. This market has “decided” that coaches who plan and prescribe training and who primarily interact through web platforms constitutes coaching. We reiterate that we believe, from a CR perspective, that sports coaching cannot be reducible to simple description, explanation (7) or definition. However, the suggestion from Cooper and Allen (89) that coaching involves social interaction involving at least two people, a coach and an athlete, and is usually focused on performance enhancement, infers that coaching using web technology is coaching.

**Table 6:** Important, interesting, and prepared by training.

Area	Ranking scores		
	Important	Interesting	Prepared for by coach education
Planning and prescribing endurance training	78	62	57
Injury prevention	73	61	27
Recovery strategies	73	65	36
Using research and sports science to inform my coaching practice	72	65	32
Using training metrics such as functional threshold power and training stress scores	70	56	31
Using online and desktop coaching software	67	54	9
Strength and conditioning	61	59	31
Mental/psychological training and preparation	59	55	25
Face-to-face technical/skills coaching	58	44	42
Technology and innovation (equipment, aerodynamics, hydrodynamics, clothing)	49	68	16
Nutrition monitoring, analysis and guidance	46	60	17
Tactical training	45	44	19
Face-to-face fitness coaching	36		33
Managing coaching programme/squad	34	35	13
Life coaching	29	21	−2
Liaising with stakeholders, other coaches and sports scientists.	24	35	−3
Holistic practices such as yoga and Pilates	23	19	−15
Team building and cohesion	20	18	4
Marketing my coaching services? (business)	19	39	−14
Talent identification and selection	4	13	−7

Furthermore, technology such as GPS, power meters and online coaching platforms have allowed the PRE-coaching market to grow. Technology now presents a threat to this coaching market in which artificial intelligence (AI) algorithms are already being used to analyse data and to design organic training programmes (98, 99). In the present study, non-coached athletes stated that cost was the greatest barrier to being coached. However, Fister et al. (98) suggests AI can overcome this barrier, in which those want more can augment AI with a “real” coach. Interestingly, their algorithm was built using readily quantifiable biophysical components of performance. Whilst it seems unlikely that such AI can build the type of interpersonal relationships that contribute to coaching effectiveness, our study suggests that many coaches and athletes do not value such interpersonal relationships as much as qualitative data driven coaching. Rather, the wider coaching market in endurance sports is technology driven and more dependent on athlete “wants” rather than an idealistic vision of what constitutes effective coaching. Therefore, the sporting discourse must be shaped through promoting a more complete coaching process and psychosocial elements of performance.

In the qualitative section of this study, coaches were asked to describe what they thought their coaching role involved. As shown in Table 7 the coaches presented a wider perspective of their role. This was reflected in the themes relating to athlete outcomes and where the coaching role dominated, suggesting that many coaches recognise a more complete coaching process. This highlights that coaches want to help athletes actualise their goals and provide a service of value. In terms of motivation in a “cottage industry”, self-satisfaction through helping others is very important. However, they must also generate income through attracting and retaining clients in sufficient numbers. Therefore, having a limited physical coach-athlete interfaces and using digital technologies may bring practical efficiencies to the coaching process, especially as work-life balance was reported to be a challenge for many coaches. However, Johnson (100) suggests that similar instructional technologies in an educational environment change power dynamics, in which the person who “pays the bill” is the figure of authority. In a digital environment where the age of athletes may be similar to their coach, such a power dynamic may not exist in the first place. Rather, athletes can “hide” in a virtual environment, deciding if and when they wish to provide feedback and what type of coaching service they want.

**Table 7:** The role of endurance coaches (with occurrences presented in parenthesis).

Higher order themes	Coaching process (42)	Coaching role (90)	Communication (7)	Education and teaching (38)	Outcome (96)	Philosophy (31)	Relationship (15)
Lower order themes	Decision maker (1)	Guide (26)	Communication (5)	Education and Teacher (22)	Achieve Potential (11)	Fun and Enjoyment (4)	Listening (4)
	Monitor and analyse (3)	Leadership (5)	Feedback (2)	Knowledge facilitation (2)	Athlete development (8)	Health promotion (8)	Partnership and Collaboration (6)
	Needs analysis (1)	Life Coaching (13)		Knowledge Transfer (13)	Goal actualisation (48)	Helping and giving to others (19)	Relationship building (5)
	Planning and strategy (14)	Mentoring (13)			Habit development (1)		
	Skill development (2)	Role Model (1)			Performance Enhancement and Optimisation (19)		
	Training Prescription (21)	Support (10)			Physical Adaptation (5)		
		Motivator and psychologist (22)			Injury prevention (4)		

### 4.3. Exploration of the digital world on coach learning

It is common for studies to recommend that coaches require better training and resources to enhance their coaching practice. However, such recommendations are based on the assumption that coaches value and engage with mediated learning experiences. Rather, we suggest that learning of endurance coaches typically occurs beyond discrete Panopticons and is often driven by web technology. This is resulting in a “total pedagogisation of society” as described by Bernstein (101) which does not respect international boundaries or the central control of institutions. In such a fluid and inter-connected digital network, simple and effective interventions to deal with complex pedagogical problems are unlikely to exist. This study shows that global brands and commercial sources dominate the information choices made by coaches. Traditionally recognised providers of coach education such as NGB's and evidence-based academic sources continue to influence the wider discourse in endurance sport (27). However, their content is not necessarily context specific or reflective of the coaching process of endurance coaches. Rather, the wider discourse is focussed towards selling tangible products, intangible services and is biophysically biased, thus neglecting more factors relating to coaching and performance enhancement. Whilst unmediated learning suggests a relative degree of learning autonomy, there appears to be an illusion of choice in what coaches want to learn about. Therefore, influencing and working with influential “brands” who present what learning choices are available to coaches may be more effective than developing learning strategies for individual coaches (102). If they recognise that they have vested interests in promoting more effective coaching, such brands could become mechanisms for change. Idealistic conceptualisations of the coaching process are unlikely to be helpful in this regard. Rather, shifting the context specific biophysical bias towards a more holistic view of performance enhancement which has social connection at its heart may play a role in coaches being able to engage with and retain athletes as customers. Thus, they can add more value within a complex and rapidly adapting market.

In such a “datafied” coaching context, technological innovation offers valuable tools to facilitate virtual connection, particularly for planning, analysing and prescribing training (103). However, we suggest that such tools threaten deeper social connection and more holistic approaches to the coaching.

In face-to-face coaching, feedback is gathered in a complex fashion in which both coaches and athletes continually reflect in action (92) as a result of different sensory inputs and outputs. This is reflected in the traditional coaching process models such as Franks et al. (104) and Magill (105) and concepts such as “evaluative” and “corrective” feedback (106). This is different in the remote environment because a coach is often not present with their athlete during training or competition. Without sensory feedback “collected” by the coach (for example through observation) and with less opportunities for the athlete to immediately express their own “intrinsic feedback” (107) coaches and athletes need to develop a range of feedback mechanisms to develop effective feedback loops, typically using digital technology. Limitations and opportunities of different digital communication mediums are discussed elsewhere (108, 109).

Remote coaching reframes the coaching process from the traditional instruction, motivation and feedback techniques based on “in-action” reflection, towards a greater focus on reflecting “on action” (92). This changes the social dynamic in the coach-athlete relationship in which is more reliant on web coaching platforms. Such communication may be augmented through other digital technologies such as WhatsApp, Messenger or text messages. More traditional face-to-face coaching inter-personal behaviours are critical to developing trust and connectedness (110, 114). We also suggest that because remote coaching is a relatively new phenomenon in endurance sport, many coaches will not have experienced being coached themselves in this environment. Furthermore, the biophysical bias within the context could mean that many coaches lack the knowledge and experience to create effective social relationships with their athletes.

**Figure 2:**
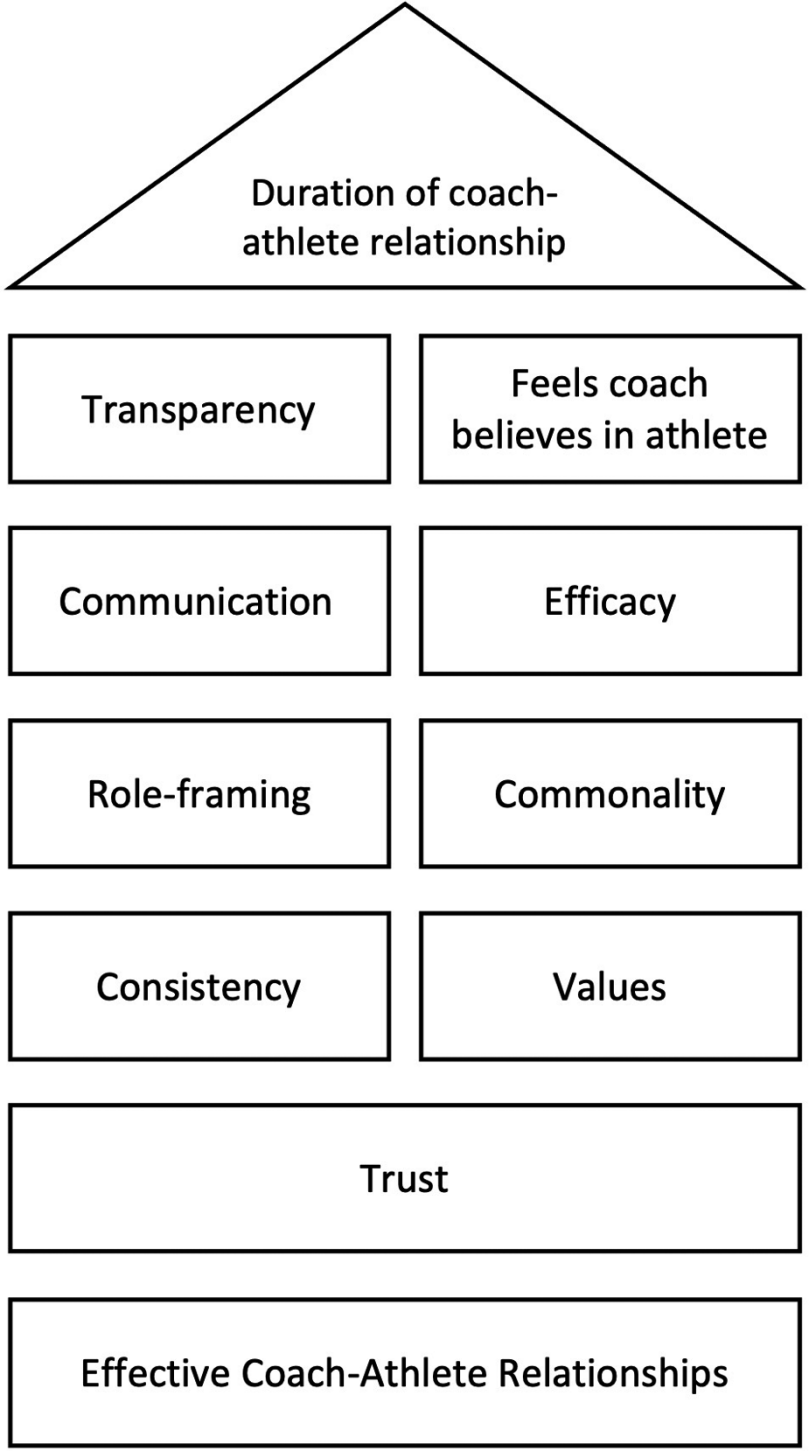
Social factors which underpin effective coach-athlete relationships in remote coaching.

Moreover, a coach-athlete relationship conducted *via* the web may result in a sense of psycho-emotional detachment in which capacity for sophisticated interpersonal interaction is limited. If this is the case, there are likely to be negative implications which limit the learning of the coach and the performance of the athlete. From a CR perspective, description of context is important in guiding future research to explore cause and causal assumptions. We suggest that an important focus of such research is to explore social connections between coaches and athletes in remote coaching environments. Preliminary findings by Britton (115) suggests that the duration of the coach-athlete relationship is a good proxy of coaching effectiveness and that themes relating to social connection as shown in Figure 2 are key.

Coach learning is often presented as something to be facilitated rather than a complex process of social osmosis in which coach behaviour is shaped by their environment (112–115). This is reflected in our data in which coaches' value unmediated learning experiences such as learning through experience and self-reflection (Table 8) more than mediated ones (14). In additional to the high-level of educational attainment mentioned previously, 86% of coaches have a coaching qualification, industry certification or a combination of both (National Federation 64%; Sports industry 21%; University 6%; Ironman University 8%; TrainingPeaks University 1%). Interestingly, 28% of triathlon coaches had an Ironman University certification. Critics of this certification, which is built upon a closed-system biophysical model, suggest that it adds little to other qualifications. However, certification allows coaches to associate themselves with and use the brand of a global industry leader which can be perceived to be helpful in the marketing of their coaching services. This finding is important because the certification was only introduced four years prior to data collection, demonstrating rapid growth, and highlighting the importance of brand in engagement.

**Table 8:** Rating of importance of the following sources of information in developing coaches practice.

Method of learning	Ranking score
Learning through experience	88
Experiences as an athlete	74
Reflecting on past coaching	73
Academic literature	64
Advice from other coaches	61
Working with/observing other coaches in your sport	61
Own experiences of being coached	59
Education outside coaching	56
Books and magazines	55
Formal coach education and qualifications	52
Websites	49
Working with/observing coaching from other sports	47
Coaching CPD, conferences and workshops	47
Being mentored	47
Watching videos	47
Experience at work outside coaching	42
Experience of being a parent	11

Werthner and Trudel (14) suggest that cognitive structure, a network of knowledge, feelings and emotions, influences what coaches pay attention to and learn from. This suggestion is not inconsistent with Hattie and Donoghue's (2) “skill, will and thrill” model of learning whereby, establishing what the learner currently knows. The data collected within this study has been interpreted to suggest that that wider sociocultural and economic milieus in endurance sport influence the type of information coaches implicitly or actively consume. The influential work of Werthner and Trudel (14) identified three types of learning situations: mediated, unmediated and internal. However, we suggest that it is not the situations or sources that are important per-se, but rather is the quality of resources that emanate from them and how they shape coaching practice. In the present study, coaches were asked to provide examples of sources of information they “paid attention to” as shown in Table 9. Coaches presented a broad array of primarily digital sources, including websites and magazines. These tended to be commercial in nature, aimed at a general endurance sport audience rather than a specific coaching population. The sources in Table 9. suggest that endurance coaches' knowledge is constructed through multiple cognitive biases towards “shiny things” (116) and is biophysically orientated. Brand is important, whether that of individual “gurus”, NGB's or commercial entities, suggesting a potential susceptibility to authority bias. Basic content analysis of these sources suggest that content is often designed to promote “clicks” or to sell products and that quality of material is highly variable. NGB and associations and industry brands and “gurus” all present similar content e.g., equipment reviews, nutrition articles, training plans/methodologies, elite athlete stories and “quick fixes”. This is presumably because efficacy can only be judged through web analytics and what proves to be popular. We suggest that such engagement is likely to influence future content more so than how effective it is in enhancing learning. This has important implications to coach education. For example, NGB coach education programmes in the UK have key performance indicators relating to sustainability and income generation. This is likely to result in mediated learning events being made “more accessible” in “bite-sized chunks”. Income will primarily be generated by content that coaches will engage with, independent of whether it will make them a better coach or not.

**Table 9:** Sources of information and knowledge for endurance coaches (*n* = 829 coaches; 1,640 sources identified; 36 responses not able to code) (occurences of mentions of each of the sources in parenthesis).

Theme	Main sources	Notes
Blogs (12)		General and non-attributable blogs
Books (90)	Joe Friel (44)	Note that Friel content relates to both books and web-content.
	Not specified (40)	
	Other (6)	
Industry brands (254)	TrainingPeaks (188)	
	Peaks Coaching (9)	
	ACE Fitness (18)	
	Ironman (5)	
	Human Kinetics (5)	
	Other (29)	
Magazines and related websites (519)	Triathlete (105)	
	Runners World (49)	
	Lava (33)	
	Running Times (28)	
	Slowtwitch (23)	
	Bicycling (12)	
	220 Magazine (11)	
	Other (258)	
Industry “Gurus” (84)		
NGB’s and Associations (260)	USA Triathlon (122)	
	American College of Sports Medicine (39)	
	USA Cycling (25)	
	British Cycling (10)	
	Other (64)	
Online Communities and Forums (28)		
Online courses (2)		
Peer Review and Scholarly (242)	PubMed (38)	Note that the majority of named journals related to physiology and the biological sciences (biophysical). Several coaches also stated that they only accessed papers in abstract form.
	Journal of Applied Physiology (11)	
	Journal of Strength and Conditioning (11)	
	Not specified or other (182)	
Strength and Conditioning Organisations (30)		
Twitter (22)		
Webinars (24)	USAT webinars (10)	
	Other (14)	
YouTube (17)		

The use of academic journals was prevalent as a learning source within the study; however, there was also an obvious biophysical bias in the sources used such as Pubmed and the Journal of Applied Physiology. Sport psychology and coaching periodicals were noticeable by their absence, despite a psychology dominant perspective within coaching practice literature (61). This suggests influences from a wider discourse in endurance sport in which there is a bias towards readily quantifiable and controllable factors and avoidance of psychosocial factors (60, 117).

The bias continued into what coaches perceived to be important, in which the top-5 highest ranked factors in Table 6 were biophysically related too. Whilst, technology and innovation were mid-ranked in terms of coaching importance, coaches found this area to be most interesting. This may reflect the fact that these relate to tangible “shiny” goods (55) that are marketed extensively in the sources where coaches access information. Whilst some sources use evidence-based content, many more do not. Although coaches felt relatively well prepared by their training to plan and prescribe training, they felt unprepared to use coaching software, in how to prevent injury, using research and training metrics by their training. They typically believed that planning and prescribing training was the most important aspect of their coaching role, with them prioritising biophysical training metrics above mental and psychological aspects of performance. This is consistent with the views of Kiely (60) in which deterministic models are used to periodise training, whilst neglecting less readily quantifiable aspects of performance. Such biases are likely to distort coaches' views on the coaching process. For example, whilst many PRE-endurance athletes experience negative psychological stressors related to training and competition (81) and would value additional support from their coach in this regard, data from the present study suggested that coaches could do a better job in this area. In a follow-up study, McCormick et al. (47) stated that dissemination of relevant, simple, concise and contextualised psychological knowledge to coaches may benefit their practice. However, if coaches perceive psychology to be less important and less interesting than physical components of performance, then they are unlikely to engage even if they will become a better coach as a result. Therefore, it is recommended that “disciplinary” researchers are cautious about recommending coaches need further education in a particular area e.g., psychology, anti-doping, nutrition without firstly considering where the subject fits within context specific coach interests.

Perhaps, a slow process of evolution rather than revolution is likely to result in learning and behavioural change (118). Promoting such evolutionary processes through research requires not only a deep understanding of the context of learning, but it also requires alternative research methods to understand stakeholder needs and buy-in (61, 81). For example, the scientific community has been slow to validate or critically evaluate Allen and Coggan's (119) Functional Threshold Power (FTP) and its associated metrics (see Table 6). Despite this lack of scientific evidence, these metrics are an important part of the coaching process relating to planning, prescribing and analysing endurance training. This may be because the principles of FTP have been integrated into endurance sport hardware such as GPS computers, cycling ergometers and then used in the analysis of data in market-leading software such as Strava, TrainingPeaks and Zwift. FTP is often discussed in quasi-scientific terms, has been extensively adopted by endurance “gurus”, professional athletes and the wider media. Methods with a greater scientific evidence-base are available (120). However, for coaches to want to learn and adopt them into practice would require a shifting of shared customary beliefs about training, software would have to be able to accommodate different coaching practices and there would have to be financial advantage to those who hold power over the discourse (121). Doing so is probably unrealistic.

Crucial to the learning of coaches is their ability to be able to interpret information from a vast array of sources. There are endless unmediated learning opportunities with information of varying quality. Whilst learning is very challenging to measure, it is a neutral process in which coaches must think and/or act differently for learning to occur. Moon (13) suggests there is a tendency to “regard learning as happening only when the learning is in a direction that is approved or desired”. Although it is useful to know where coaches access their information from, it is more important to recognise these sources are mere representations of the knowledge of others. Further, such knowledge is often biased by vested interests, the need to sell something or presented using editorial styles designed for information consumption. That is not to say that some of the sources may not contain credible content. Rather, for learning to occur, coaches must be sufficiently intellectually skilled to ensure they do not simply accept information as true or to blindly accept advice provided without deeper cognitive processing. In other words, they need to be critical consumers of information (126). The study of Koh et al. (127) is one of a very few to have explored the use of the internet as a learning source for coaches, in a youth soccer context. These coaches appeared to access similar types of sources which they deemed to be credible, including federations, high-profile clubs and coaches. They also used their experiences as players as a frame of reference to decide if the information was valuable to their coaching practice or not. Koh et al. (127) suggested that discerning the credibility of the sources was a challenge as was coaches primarily engaging with material that confirmed pre-existing beliefs. Our data suggests that academic attainment does not offer enough protection against such bias, in which self-sourced and random browsing results in coaches engaging with information that is consistent with the wider discourse. Rather, powerful forces push them towards assimilation with market driven agendas and a learning status-quo.

### 4.4. Limitations

Whilst this study has contributed to knowledge of learning in the context of endurance sport, no research is without limitations. It is recognised that this research was part of a wider exploratory project seeking to better understand the coaching practice and learning of endurance coaches. This is a critical part of the research process in which the purpose is to “identify real-world problems and issues that athletes and coaches face” (72). Consistent with the recommendations of those who advocate methods to promote impact [e.g., (62, 72, 77)] we engaged beyond the research community. Such an approach came with several challenges. Firstly, holding together a tightly framed and cohesive narrative whilst retaining the complex nature of the coaching environment was highly challenging. This paper /study presents an evidence guided and reflexive view from a sports coaching /coach's perspective. Therefore, there is no claim made that the research is free from bias. However, an important role of the second author was to “check and challenge” interpretation. The study narrative attempts to tackle complexity in a broad CR conceptualisation of endurance coaching practice (61) exploring potential interacting factors which influence causation. This includes considering the interaction between multiple stakeholders and actors. The desire was not to provide a new conceptualisation of coaching but to shift the orientation of thinking of the reader (13). A more tightly focussed “objective” lens may have resulted in something that was easier to grasp but it may have failed to capture the essence of the coaching context and been of little practical effect (123). Secondly, as the main market for coaches, we chose a PRE-focussed dialogue, whilst potentially diverting analyses from other segments such as ERE, youth-development and recreational sport. However, these segments have been explored extensively and were less likely to add new knowledge or debate surrounding endurance sport. Furthermore, comment on the manuscript was sought from industry professionals prior to submission and there was broad consensus that it offered a fair representation of context.

Whilst there are commonalities with the coaching process described by Cooper and Allen (89) in which coach-athlete interactions shape learning, no attempt was made to present a unified theory of coach learning or the coaching process. However, it would be interesting to explore this process using more in-depth qualitative approaches. Finally, because of the way participants were recruited, a sampling bias may have occurred in which participants using digital technology were more likely to have completed the survey (124). We attempted to overcome this limitation by engaging with major NGB's to help promote the survey with their members. However, even with strong links to these organisations, there was an apparent reticence by some to assist. Within the UK, we suggest that such reticence may be influenced by the fact that focus of coach development programmes is often driven by rhetoric-based policies rather than evidence-based needs of NGB member coaches.

## 5. Conclusions and future directions

The findings of the study provide valuable insights into the coaching context of endurance coaches, what they do and how they learn. Through presenting a broad narrative, our results illustrate a complex relationship between how coaches and athletes interact in an environment shaped by technology and market forces. We also suggest that NGB's are constrained by national boundaries and the agendas of those who funds them in the provision of coach education. Furthermore, unmediated learning sources are more influential than mediated learning provided from within such Panipticons. Therefore, in line with the recommendations of Stoszkowski et al. (127) we suggest that mediated coach education should support coaches to filter the noise within their coaching context. To do so requires critical thinking skills, independent confirmation of the facts and the recognition that the answer to most coaching questions is “it depends”. Endurance coaches must also learn to recognise the biophysical biases in their practices and an apparent myopia towards psychosocial elements of coaching. Further empirical studies exploring the influence of unmediated learning sources on coaching practice may be merited. Doing so could give broader insight into how people learn in the digital age. Further investigations to explore how coach-athlete relationships develop using web-technology and what influence they have on performance is also merited.

## Data Availability

The raw data supporting the conclusions of this article will be made available by the authors, without undue reservation.
